# Cysteine String Protein Controls Two Routes of Export for Misfolded Huntingtin

**DOI:** 10.3389/fnins.2021.762439

**Published:** 2022-01-05

**Authors:** Desmond Pink, Julien Donnelier, John D. Lewis, Janice E. A. Braun

**Affiliations:** ^1^Nanostics Precision Health, Edmonton, AB, Canada; ^2^Department of Biochemistry and Molecular Biology, Cumming School of Medicine, Hotchkiss Brain Institute, University of Calgary, Calgary, AB, Canada; ^3^Department of Oncology, University of Alberta, Edmonton, AB, Canada

**Keywords:** molecular chaperone, JDP, DnaJ, microflow cytometry, Huntington’s disease, export

## Abstract

Extracellular vesicles (EVs) are secreted vesicles of diverse size and cargo that are implicated in the cell-to-cell transmission of disease-causing-proteins in several neurodegenerative diseases. Mutant huntingtin, the disease-causing entity in Huntington’s disease, has an expanded polyglutamine track at the N terminus that causes the protein to misfold and form toxic intracellular aggregates. In Huntington’s disease, mutant huntingtin aggregates are transferred between cells by several routes. We have previously identified a cellular pathway that is responsible for the export of mutant huntingtin *via* extracellular vesicles. Identifying the EV sub-populations that carry misfolded huntingtin cargo is critical to understanding disease progression. In this work we expressed a form of polyglutamine expanded huntingtin (GFP-tagged 72Qhuntingtin^exon1^) in cells to assess the EVs involved in cellular export. We demonstrate that the molecular chaperone, cysteine string protein (CSPα; DnaJC5), facilitates export of disease-causing-polyglutamine-expanded huntingtin cargo in 180–240 nm vesicles as well as larger 10–30 μm vesicles.

## Introduction

The cell-to-cell transfer of extracellular vesicles (EVs) is a conserved process. *In vivo*, the continuous exchange among different cells generates a dynamic and heterogeneous pool of EVs (Pegtel and Gould, 2019). EVs come in different sizes and carry different cargoes that exert profound effects in recipient cells following uptake. The physiological roles of EVs include exchanging information between cells as well as removing unwanted proteins from cells (Vella et al., 2016; Hill, 2019; Pegtel and Gould, 2019). EVs are also implicated in disease progression, however, their role is far from clear. How EVs facilitate the spread of disease in cancer and neurodegenerative disease and what distinguishes physiological from pathological EVs is a current focus of investigation (Hill, 2019; Pegtel and Gould, 2019). While complex cargoes of DNA, RNA, proteins, lipids, and metabolites are packaged in EVs for delivery to recipient cells, consensus has not yet emerged on specific markers of EV subtypes (Thery et al., 2018) and our understanding of the mechanisms that target proteins to EVs is rudimentary in comparison to conventional secretion. Moreover, EVs arise from multiple subcellular origins and, in many instances, the particular pathway is not known (Thery et al., 2018). The most widely studied EVs are exosomes, which originate from fusion of multivesicular bodies with the plasma membrane and microvesicles, which form from outward budding of the plasma membrane. The function of other EVs such as exophers, which export misfolded cargo from viable cells, is only beginning to be unraveled (Melentijevic et al., 2017; Nicolas-Avila et al., 2020). EVs are widely considered potential disease biomarkers (Hill, 2019), particularly peripheral EV that carry misfolded-pathogenic protein cargo such as prions (Fevrier et al., 2004; Vella et al., 2008), beta-amyloid peptides (Rajendran et al., 2006), amyloid precursor protein fragments (Sharples et al., 2008), tau (Stern et al., 2016), mutant superoxide dismutase-1*^G93A^* (Gomes et al., 2007; Grad et al., 2014a,b), TDP-43 (Nonaka et al., 2013), α-synuclein (Emmanouilidou et al., 2010; Alvarez-Erviti et al., 2011), and polyglutamine expanded huntingtin (Deng et al., 2017).

Trinucleotide repeat expansions of the huntingtin gene cause Huntington’s disease, a devastating progressive neurodegenerative disorder that manifests in midlife (Gusella and MacDonald, 2000). Aggregates of polyglutamine-expanded huntingtin are found within genetically normal tissue grafted into patients with progressing Huntington’s disease, indicating cell-to-cell transit of huntingtin aggregates *in vivo* (Cicchetti et al., 2014). Molecular chaperones and co-chaperones are promising candidates for the treatment of neurodegenerative diseases like Huntington’s disease. To date, the majority of studies conducted to evaluate and modify the toxicity of mutant huntingtin have focused on the intracellular molecular chaperones that suppress aggregation and/or promote degradation of misfolded protein (Howarth et al., 2007; Hageman et al., 2010; Gillis et al., 2013). Here we focus on cysteine string protein (CSPα; DnaJC5), an abundant molecular co-chaperone in neurons that facilitates export of mutant huntingtin in EVs (Deng et al., 2017).

CSPα is a member of the J domain containing protein (JDP) family that contains a unique string of cysteine residues (Braun and Scheller, 1995). The human genome encodes 53 JDPs that deliver misfolded protein substrates to Hsp70 and activate Hsp70ATPase activity through a conserved histidine, proline, aspartate (HPD) motif (Kampinga and Craig, 2010). As key regulators of the cellular Hsp70 machinery, JDPs are critical to protein homeostasis. In particular, CSPα is a highly conserved presynaptic JDP that makes it possible for synapses to keep running for extended time periods (Zinsmaier et al., 1994; Fernandez-Chacon et al., 2004). There are a number of possible mechanisms by which JDPs, like CSPα, might maintain synapses, including the refolding of misfolded proteins, the directing of misfolded proteins for intracellular elimination by proteosomes or lysosomes or the export of toxic proteins. In this work we performed unbiased analysis of the export of aggregated huntingtin from a cell culture model in order to explore which EVs carry toxic huntingtin. Using microflow cytometry and live cell imaging we establish that two subpopulations of EVs, a smaller physically sized population in the range of 180–240 nm silica and a larger 10–30 μm, are responsible for the CSPα-mediated export of mutant huntingtin. Furthermore, we show that mutant huntingtin export can be pharmacologically modified. Specifically we show that resveratrol, a polyphenol initially identified as a modulator of the CSPα pathway in a *C. elegans* screen (Kashyap et al., 2014), reduces misfolded huntingtin export through both EV export pathways.

## Materials and Methods

All experimental protocols were approved by the University of Calgary or University of Alberta. All experiments were carried out in accordance with the relevant institutional guidelines and regulations.

### Extracellular Vesicle Collection From CAD Cells

**FIGURE 1:**
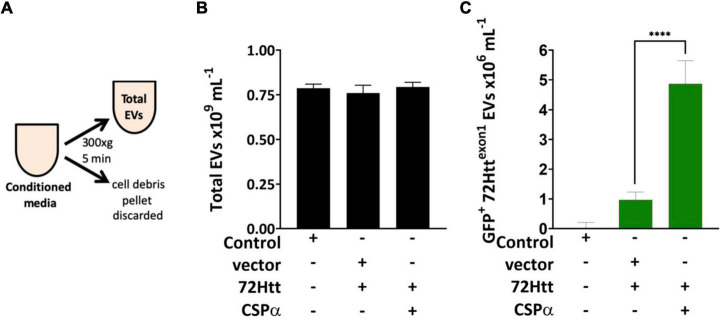
Microflow cytometry showing the influence of CSPα on EV export **(A)** experimental approach, EVs were examined without enrichment. **(B)** Bar graph of the concentration of total EVs released and **(C)** bar graph of the GFP-72Htt^exon1^-containing EVs released as determined by microflow cytometry (****P < 0.0001).

**FIGURE 2:**
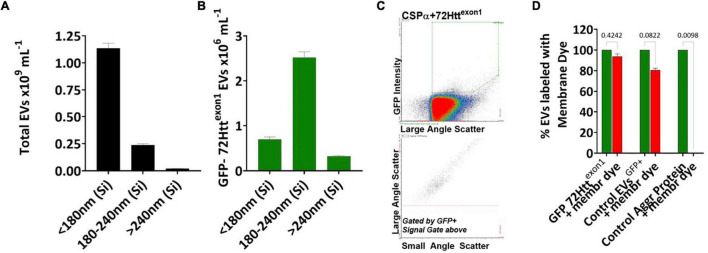
CSPα export of EVs in the 180–240 nm silica range Bar graphs showing the sizes of the **(A)** total EVs and **(B)** GFP-72Htt^exon1^ containing EVs, Silica (Si). **(C)** Flow cytometry scatter plot of GFP-72Htt^exon1^ containing EVs. **(D)** EVs containing GFP-72Htt^exon1^, control EVs containing palmitoylated GFP and the control aggregated protein, anti-CD36 FITC, were stained with Deep Red membrane stain. The experiment was replicated 10–12 times, *p*-values are indicated.

**FIGURE 3:**
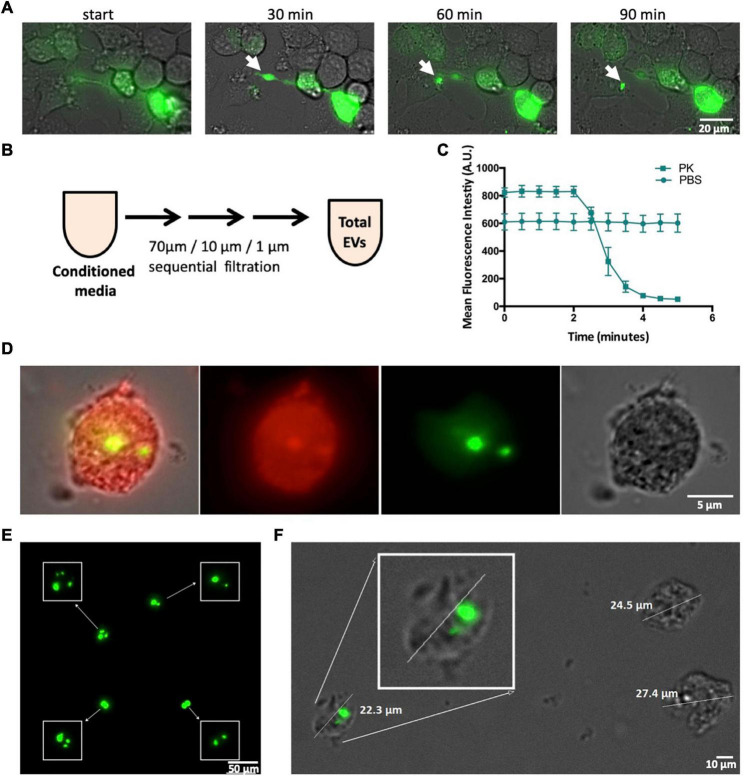
CSPα export of 10–30 μm EVs. **(A)** Widefield time lapse fluorescent microscopy with brightfield of EV export containing GFP-tagged huntingtin aggregates from a donor cell. **(B)** Experimental approach, EVs were examined following filtration. **(C)** EVs were treated with 0.01 mg/ml proteinase K () or PBS () and fluorescence monitored over time. **(D)** Deep red membrane stain of an EV containing GFP-tagged huntingtin aggregates. **(E)** High-resolution, fluorescence deconvolution microscopy of GFP-tagged huntingtin aggregates located in EVs. **(F)** Size of EVs exported from CAD cells.

**FIGURE 4:**
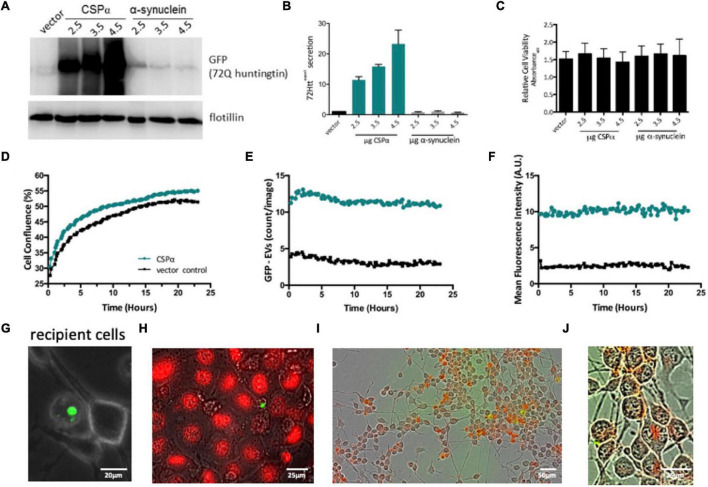
Characterization of EVs. **(A)** Western analysis of EVs collected from CAD cells expressing GFP-tagged 72Q Htt^exon1^ and CSPα or α-synuclein (control protein). Blots are probed for GFP and flotillin. Western blot is representative of 4 independent experiments. Flotillin is shown as a loading control. **(B)** Bar graph quantifying western analysis of exported GFP-tagged 72Q Htt*^exon1^*. **(C)** Relative cell viability of CAD cells following media collection. **(D)** IncuCyte live cell analysis of recipient cell confluence following application of EVs to naïve cells in 6 well plates. Green represents EVs from cells expressing CSPα and GFP-mutant huntingtin, black represents EVs from cells expressing vector (control) and GFP-mutant huntingtin. **(E)** Number of large EVs containing GFP (puncta intensity = 1, rdiameter = 10 averaged over 18 images). **(F)** Fluorescence intensity of large EVs containing GFP (relative units; mean from 18 images). **(G)** Representative EVOS fluorescent image of a large EV and recipient cells. **(H)** EVs containing GFP-tagged huntingtin aggregates and naïve CAD cells were stained with DRAQ5 for DNA and imaged with widefield fluorescence and brightfield microscopy. **(I,J)** EVs (not enriched) were labeled with Deep Red stain, applied to recipient cells, incucyte images are 12 h after EV application.

**FIGURE 5:**
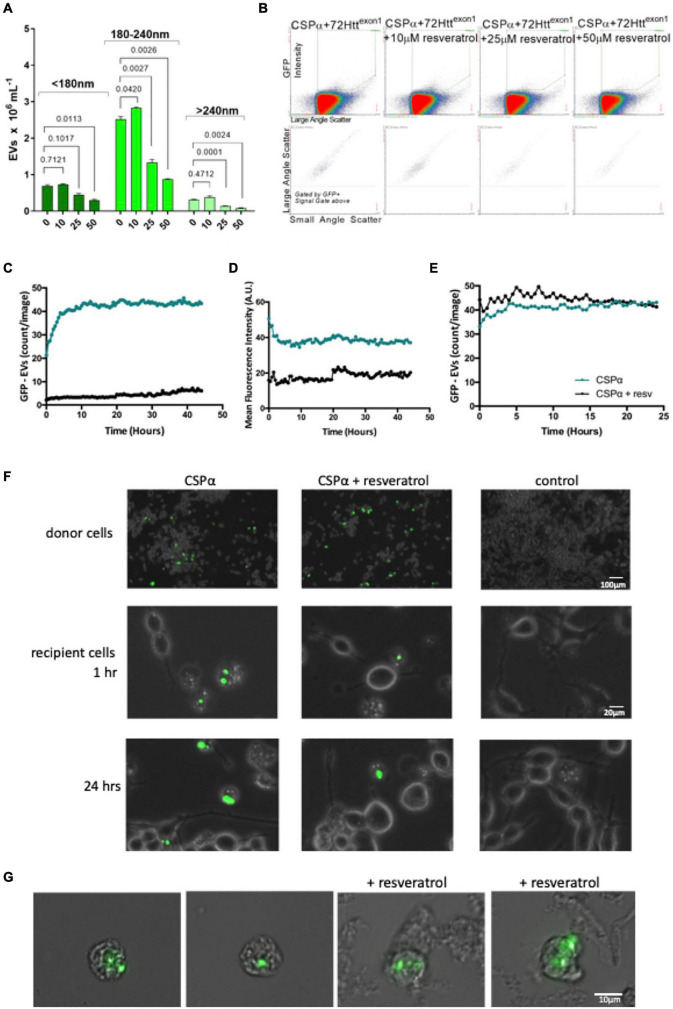
Resveratrol reduces CSPα-EV export of mutant huntingtin. **(A)** Bar graph showing GFP-72Htt^exon1^ containing EVs released in the presence of increasing concentrations of resveratrol, *p*-values are indicated. **(B)** Flow cytometry scatter plot showing GFP-72Htt^exon1^ containing EVs released in the presence of increasing concentrations of resveratrol. **(C,D)** EVs from CAD cells co-expressing GFP-mutant huntingtin and CSPα in the absence (green) and presence of 50 μm resveratrol (black) were applied to naïve cells in 12 well plates. **(C)** Number of large EVs containing GFP (puncta intensity = 1, rdiameter = 10, and mean fluorescence averaged over 9 images) and **(D)** fluorescence intensity of large EVs containing GFP (relative units; mean from 9 images). **(E)** The number of EVs expressing GFP-mutant huntingtin and CSPα were monitored for 24 h following direct application of 50 μm resveratrol. **(F)** EVOS images of GFP-72Htt^exon1^ aggregates in donor CAD cells at the time of media collection and GFP-72Htt^exon1^ aggregates in recipient cells at 1 and 24 h. **(G)** Widefield fluorescent microscopy of EVs containing GFP-tagged huntingtin aggregates.

For EV production, CAD cells (catecholaminergic derived CNS cells) were seeded in 10 cm culture dishes in Dulbecco’s Modified Eagle’s Medium (DMEM-F12 Gibco Thermo Fisher Scientific), supplemented with 10% Fetal Bovine Serum (FBS, Gibco Thermo Fisher Scientific), 1% penicillin (100 U/ml) and streptomycin (100 μg/ml) (P/S, Gibco Thermo Fisher Scientific) and maintained at 37°C, 5% CO_2_ atmosphere. After 24 h cells were transfected with indicated plasmids using Lipofectamine 3000 (Invitrogen) in Opti-MEM™ medium. 6 h after transfection, medium was changed to serum free Dulbecco’s Modified Eagle’s Medium (DMEM Gibco Thermo Fisher Scientific), supplemented with 1% penicillin and streptomycin. Conditioned medium was collected 48 h after transfection and spun at 300 × g for 5 min to remove cell debris and larger particles and then evaluated by microflow cytometry analysis and live cell imaging (Figures 1 and 2). Following media collection, cell viability was determined utilizing an XTT assay (New England Biolabs). Similar results were obtained utilizing calcium phosphate transfection methods. Where indicated media was processed by sequential filtration through 70 μm (nylon) filters followed by 10 μm/1 μm (PET; Polyethylenterephthalat) filters (pluriSelect) with negative pressure applied *via* a syringe (Figures 3–5).

### Immunoblotting

For western analysis following the 300 × g spin EVs were precipitated and solubilized in sample buffer. Proteins were separated by SDS-PAGE and electrotransferred from polyacrylamide gels to nitrocellulose membrane (0.2 μm pore size). Membranes were blocked in tris-buffered saline (TBS) containing 0.1% Tween 20, 1% BSA and then incubated with primary antibody overnight at 4°C. The membranes were washed and incubated with horseradish peroxidase-coupled secondary antibody for ∼2 h at room temperature. Bound antibodies on the membranes were detected by incubation with Pierce chemiluminescent reagent and exposure to Cdigit, LiCor (Mandel). The chemiluminescent signals were quantified using image studio digits software (Mandel).

### Plasmids

cDNAs encoding for CSPα, CSPα mutants, and α-synuclein were expressed in the plasmid myc-pCMV. GFP-72Q huntingtin^exon1^ was expressed in the plasmid pcDNA3.1. All amplified regions of all plasmids were sequenced to ensure the absence of any undesired mutations.

### Fluorescence Imaging

Nikon widefield images were acquired at room temperature on a Nikon Ti Eclipse widefield microscope equipped with a Hamamatsu Orca flash 4.0 v2 sCMOS 16-bit camera using NIS-Elements AR v5.00.00 64-bit software. Images were captured using either a 40x Plan Apo λ/0.95 numerical aperture (NA) objective or a 60x Plan Apo λ/1.4 NA oil objective.

Incucyte images were acquired on an Essen BioScience IncuCyte Zoom microscope using IncuCyte Zoom v2018A software. The IncuCyte Zoom microscope was in a humidified incubator at 37°C with 5% CO_2_. Nine images per well of either six-well plates (Figure 4) or 12-well plates (Figure 5) were captured every 15 min for 24 h using a 20x Plan Fluor/0.4 NA objective.

Analysis was performed using IncuCyte Zoom v2018A software. Analysis masks were created to determine confluency (confluence mask) and presence of green objects (green object mask). These masks were applied to all images in a data set.

EVOS FL auto images of live donor cells were taken at the time of media collection. Images of recipient cells were taken between 1 and 24 h. The intensity of the GFP-72Q Htt^exon1^ varied and we used the single slider until the on-screen brightness of the lowest intensity aggregate was satisfactory and used this setting to capture images in high-quality mode.

### Microflow Cytometry

Particle size and concentration of the samples were determined *via* microflow cytometry using Apogee A50 flow cytometry platform. Samples were similarly prepared for NTA and Microflow cytometry. Light scatter was provided using the 405 nm laser (75 mW); GFP signal was generated using the 488 nm laser (50 mW, 535/35) and far red signal was generated using the 630 nm laser (75 mW, 680/35). All samples were analyzed for 60 s. Optimization was performed to insure single EVs were being analyzed and single events were triggered by light scatter only. The system was cleaned each day prior to sample analysis and a variety of silica and polystyrene standards (Apogee 1493 standards) processed for instrument set up and monitoring. The silica standards were used to assess the relative size range of EVs (Supplementary Figure 1).

To determine if the GFP-72Q Htt^exon1^ signal was associated with vesicles and not simply protein aggregates, samples were first mixed with Cell Mask Deep Red plasma membrane stain (Thermo Fisher Scientific; C10046), final concentration of 0.1X), incubated for 30 min at 37°C and then diluted in PBS. For comparison, palmitoylated-GFP positive EVs were obtained from the conditioned media of a PC3 prostrate cancer cell line; the membrane of these EVs contain GFP proteins. Ten microliter of anti-CD36-FITC (IgG) antibody was incubated at 50°C for increasing periods of time to generate thermally induced non-membrane, protein aggregates (technique based upon Malvern Pananalytical application note AN140303 *Aggregation in Proteins*). The protein aggregate controls [anti-CD36-FITC (IgG)], as well as the palmitoylated-GFP EV controls, were similarly stained with Deep Red stain. Aggregate concentration was analyzed by NTA and uptake of the membrane dye was measured using microflow cytometry. For these microflow cytometry experiments, single particle detection was triggered using positive GFP/FITC fluorescence and the associated far red signal analyzed.

### Statistical Analysis

All data were graphed and statistically analyzed using GraphPad Prism version 6.01 for Windows, GraphPad Software, La Jolla, California, United States.^1^ Statistics included One-Way and Two Way ANOVA with either Tukey’s or Dunnett’s post-test analysis if initial ANOVA was statistically significant (*p* < 0.05; stars indicate significance ★ *p* < 0.05, ★★ *p* < 0.01, ★★★ *p* < 0.001, ★★★★ *p* < 0.0001). All values are presented as the mean ± SEM where appropriate, otherwise the SD is presented as indicated.

## Results

### Cysteine String Proteinα Mediates Export of Extracellular Vesicles in the 180–240 nm (Silica) Size Range

We first asked the question; Is misfolded huntingtin cargo common to all EVs or to select EVs? To address this question, media from CAD neural cells expressing CSPα and GFP-tagged 72Q huntingtin^exon1^ was collected and centrifuged at 300Xg for 5 min to remove cell debris (Figure 1A) and EVs evaluated by microflow cytometry (Maia et al., 2020). We used minimal processing of the conditioned media to permit analysis of the broadest spectrum of EV populations and avoid selection of sub-populations of EVs. While secretion of total EVs remains unchanged following expression of GFP-tagged 72Q huntingtin^exon1^ or co-expression of CSPα and GFP-tagged 72Q huntingtin^exon1^ (Figure 1B), secretion of EVs containing GFP-tagged 72Q huntingtin^exon1^ is dramatically increased when cells express CSPα (*p* < 0.0001)(Figure 1C), confirming our previous work. EVs released from CAD cells in the presence of CSPα are heterogeneous. Particles analyzed on the Apogee A50 can be resolved down to 110 nm polystyrene or just less than 180 nm silica (Supplementary Figure 1); this resolution corresponds to ∼374 nm for biological particles based on the ability to resolve the Verity Shell 01B. Small EVs (<180 nm Silica), comprised the majority of exported vesicles (81.6%) (Figure 2A). However, the bulk of EVs packaged with mutant huntingtin fall within the 180–240 nm (silica) range (Figures 2B,C). The EVs in the 180–240 nm Silica size range make up 17% of the total EVs (Figure 2A) and CSPα increased export of EVs containing GFP-tagged 72Q huntingtin^exon1^ within the 180–240 nm (Silica) EV subpopulation by > 400% (Figures 2B,C).

We next sought to determine if the GFP-72Q huntingtin^exon1^ particles detected by microflow cytometry were vesicular or non-vesicular particles. To do so, EVs released from CAD cells co-expressing CSPα and GFP-tagged 72Q huntingtin^exon1^ were labeled with Cell Mask Deep Red plasma membrane stain for 30 min at 37°C. We found that 95% of GFP-72Q huntingtin^exon1^ particles stained with Deep Red stain, demonstrating the majority of GFP particles are membrane bound vesicles and not free-floating entities (Figure 2D and Supplementary Figure 2). In comparison, 80% of EVs released from PC3 prostrate cancer cells, that express and export palmitoylated-GFP, stain with Deep Red stain. In contrast, only 0.6% of the control aggregated protein, anti-CD36-FITC, stain with Deep Red stain. Together, these results show that CSPα expression correlates with an increase in export of GFP-72Q huntingtin^exon1^ in EVs in the 180–240 nm Silica size range.

### Cysteine String Proteinα Mediates Export of 10–30 μm Extracellular Vesicles

We also found mutant huntingtin in large EVs outside of the ∼180–1,300 nm (Silica) range of microflow cytometry (Apogee A50). Figure 3A is a live cell imaging time lapse showing representative export of a large EV containing GFP-72Q huntingtin^exon1^ cargo from CAD cells reminiscent of protein aggregate export in exophers. To further examine these larger EVs, media was collected from CAD cells and submitted to sequential [70 μm (nylon) 40 μm/1 μm (PET Polyethylenterephthalat)] filtration (Figure 3B). Despite their size—large EVs were found to be pliant and pass through filters. GFP-72Q huntingtin^exon1^ aggregates within the large EVs are protinease K sensitive (Figure 3C). Figure 3D shows a large EV from filtered media that contains misfolded huntingtin cargo (green), the EV membrane is stained with Deep Red (red). Deconvolution microscopy reveals the presence of multiple GFP-72Q huntingtin^exon1^ aggregates within a single membraneous structure (Figure 3E), however, not all EVs contain huntingtin aggregates (Figure 3F). A representative EV is shown in Figure 3F, the mean size of EVs that contain GFP-72Q huntingtin^exon1^ aggregates was found to be 16.6 μm (S.D = 3.44; *n* = 81).

### Donor Cells Are Viable Following Export of Extracellular Vesicles Carrying Misfolded Huntingtin and Recipient Cells Are Viable Following Application of Extracellular Vesicles Carrying Misfolded Huntingtin

We next evaluated the connection between mutant huntingtin export and expression levels of CSPα by collecting media from CAD neural cells co-expressing vector or increasing concentrations of CSPα and GFP-tagged 72Q huntingtin*^exon1^*. Cell debris was removed, EVs were precipitated and exported GFP-72Q huntingtin^exon1^ was evaluated by western analysis. CSPα-EV export of GFP-72Q huntingtin^exon1^ increases in the presence of increasing cellular CSPα expression (Figures 4A,B) and co-expression of GFP-72Q huntingtin^exon1^ with another control protein, α-synuclein, does not result in mutant huntingtin export. Cell viability evaluated after media collection was not influenced by CSPα expression (Figure 4C), further indicating that healthy cells export GFP-72Q huntingtin^exon1^ and export is not a consequence of cell lysis.

We then assessed whether EVs carrying misfolded huntingtin were detrimental to recipient cells. Media was collected from cells co-expressing vector or CSPα and GFP-72Q huntingtin^exon1^ and cell debris removed. The heterogeneous pool of EVs, including the 180–240 nm and 10–30 μm EVs containing GFP-72Q huntingtin^exon1^, were applied to naïve recipient cells and the 10–30 μm EVs containing mutant huntingtin monitored by live cell imaging for 24 h (nine images per well were captured every 15 min). EVs originating from CSPα/GFP-72Q huntingtin^exon1^ expressing cells have more aggregates (green) compared to vector/GFP-72Q huntingtin^exon1^ control (black). No change or dysfunction in CAD cell division was observed in the presence of EVs containing huntingtin cargo, indicating the EVs are not toxic to recipient cells (Figure 4D). In order to evaluate the stability of the large EV with mutant huntingtin cargo following application to recipient cells, the number and fluorescence of huntingtin aggregates were monitored for 24 h. Direct monitoring of the 10–30 μm EVs as fluorescent puncta shows that they are stable in number (Figure 4E) and fluorescence intensity (Figure 4F) and do not undergo degradation within the 24 h time window. A representative image of a large EV with naïve recipient cells is shown in panel 4G and an image of a large EV with recipient cells stained for DNA with DRAQ5 is shown in Panel 4H. Cells are clearly DRAQ5 positive, while EVs containing GFP-72Q huntingtin^exon1^ do not stain with DRAQ5 indicating the EVs do not contain nuclear-like levels of DNA. Although the large GFP-72Q huntingtin^exon1^ containing EVs are not taken up by recipient cells, when the heterogeneous EV pool including both 180–240 nm and 10–30 μm EVs containing GFP-72Q huntingtin^exon1^ was stained with Deep Red and then applied, recipient cells show uptake of Deep Red membrane stain 12 h after EV application (Figures 4I,J). Based on these observations, it can be concluded that the huntingtin aggregates, exported in the presence of CSPα are non-toxic to cells and 10–30 μm EVs containing GFP-72Q huntingtin^exon1^ are stable.

### Resveratrol Reduces Export of Mutant Huntingtin

Resveratrol has been shown to reduce export of mutant huntingtin by CSPα (Deng et al., 2017), therefore, we sought to determine whether the 180–240 nm (Silica) EV export was affected by resveratrol. To do so, we co-expressed GFP-tagged 72Q huntingtin^exon1^ with CSPα and then treated with resveratrol 6 h following transfection and collected EVs for analysis. Figures 5A,B shows a concentration dependent resveratrol-reduction in secretion of the 180–240 nm (Silica) GFP labeled EV pool. We then asked whether resveratrol also altered mutant huntingtin export in the larger 10–30 μm EVs. EVs were collected from CAD cells 48 h following expression of GFP-72Qhuntingtin^exon1^ with CSPα, processed by sequential filtration and applied to recipient cells. Fewer large EVs with aggregates originated from CSPα/GFP-72Qhuntingtin^exon1^expressing cells in the presence (black) compared to the absence of resveratrol (green). Neither the number of EVs carrying GFP-72Qhuntingtin^exon1^ cargo or the GFP fluorescence diminishes over time, again demonstrating the stability of exported mutant huntingtin in large EVs (Figures 5C,D). Direct application of resveratrol to already exported EVs did not reduce GFP-72Qhuntingtin^exon1^ in EVs, indicating that resveratrol has a pre-EV export mode of action (Figure 5E). Figure 5F shows recipient cells after application of EVs from CSPα/GFP-72Qhuntingtin^exon1^ expressing cells treated with or without resveratrol. Figure 5G compares images of large EVs containing misfolded huntingtin from filtered media collected from CSPα-expressing cells in the absence and presence of resveratrol. Although lower in abundance, the 10–30 μm EVs are clearly detectable following resveratrol treatment.

## Discussion

We have found that CSPα mediates export of mutant huntingtin through two subpopulations of EVs, sized at 180–240 nm and 10–30 μm and that resveratrol reduces export of mutant huntingtin through both EV routes. The large EVs contain multiple mutant huntingtin aggregates which are pliable and sensitive to proteinase K degradation, yet stable when applied to recipient cells. We speculate that export of mutant huntingtin is a mechanism to maintain proteostasis in donor cells and dispose of harmful misfolded proteins to cells with greater clearance capacity for toxic protein aggregates. This would be particularly relevant in neuronal synapses where the basic proteostatic machinery is limited. Identification of two separate EV export pathways argues for the importance of export in managing misfolded protein levels, however, the exact role these two export pathways play in neurodegenerative disease progression will require further investigation.

CSPα, a synaptic JDP that significantly enhances the ATPase rate of Hsc70 through its N terminal J domain (Braun et al., 1996), directly interacts with misfolded huntingtin (Miller et al., 2003). Co-expression of CSPα and GFP-tagged 72Q huntingtin^exon1^ in cell culture models leads to the cellular export of misfolded huntingtin but not native huntingtin (Deng et al., 2017). The export of EVs carrying mutant huntingtin cargo by CSPα is dependent on the histidine, proline, aspartate (HPD) motif, located within the J domain of CSPα, revealing the requirement of an active CSPα/Hsc70 chaperone complex for export (Deng et al., 2017). In addition to mutant huntingtin, CSPα is reported to facilitate export of several different unfolded and toxic proteins known to cause neurodegeneration. Fontaine et al. (2016) have demonstrated that CSPα increases secretion of TDP-43, α-synuclein, and tau from HEK293 cells. Furthermore, Lee et al. (2018) and Xu et al. (2018) have shown that CSPα facilitates export of, TDP-43, α-synuclein, and tau from HEK and COS7 cells. We have shown that CSPα exports SOD-1*^G93A^ via* EVs as well as mutant huntingtin (Deng et al., 2017).

Consistent with its role in the export of pathogenic proteins, CSPα is not essential for synaptogenesis but is critical for the maintenance of synaptic transmission. CSPα knock-out mice exhibit neurodegeneration and have a reduced lifespan with no mice surviving beyond 3 months (Fernandez-Chacon et al., 2004). Neurons with high electrical activity such as GABAergic neurons and photoreceptors show early and prominent degeneration, suggesting that high synaptic activity potentiates degeneration (Fernandez-Chacon et al., 2004; Schmitz et al., 2006; Garcia-Junco-Clemente et al., 2010). 1-year-old CSPα heterozygous mice have a reduced neuromuscular response to repetitive stimulation compared with wild time mice, suggesting the levels of CSPα may influence the severity of neurodegeneration (Lopez-Ortega et al., 2017). Loss-of-function CSPα *Drosophila* mutants demonstrate temperature-sensitive paralysis and early lethality (Zinsmaier et al., 1994). And, in *C elegans*, CSPα null mutants display neurodegeneration and reduced lifespan (Kashyap et al., 2014). Not surprisingly, CSPα dysfunction has been implicated in several neurodegenerative disorders (Miller et al., 2003; Chandra et al., 2005; Zhang et al., 2012; Donnelier et al., 2015; Tiwari et al., 2015; Henderson et al., 2016).

Resveratrol is a known multi-target polyphenol with neuroprotective, cardioprotective and anti-inflammatory properties (Kashyap et al., 2014). We have found that resveratrol reduces the levels of pathogenic huntingtin that is exported through both 180–240 nm and 10–30 μm EV subtypes. Resveratrol was first shown in a *c. elegans* screen to ameliorate the reduced life span of CSPα mutants suggesting a role in proteostasis (Kashyap et al., 2014). It is possible that resveratrol alters the interplay between EV export and lysosomal and degradative pathways. Therapeutic agents that modify the levels of EVs carrying pathogenic proteins are of considerable interest.

Neural export of misfolded proteins offers many advantages such as maintenance of parent cell protein quality control but also many dangers if unregulated. Data presented here illustrate a dual EV export system that removes toxic huntingtin from cells and link the molecular co-chaperone CSPα to EV genesis and export. We speculate that the cell-to-cell transfer of toxic huntingtin becomes progressively dysregulated during Huntington’s disease progression. These findings highlight the dynamic nature of the proteostasis network and promising future of therapeutic strategies which target it.

## Data Availability Statement

The original contributions presented in the study are included in the article/Supplementary Material, further inquiries can be directed to the corresponding author/s.

## Author Contributions

JB conceived the project, designed, and interpreted all WB and FI experiments. DP and JL designed and interpreted all NTA and MFC experiments. JD provided technical assistance. JB, DP, and JL wrote the manuscript. All authors contributed to the article and approved the submitted version.

## Conflict of Interest

DP and JL were employed by Nanostics Precision Health. The remaining authors declare that the research was conducted in the absence of any commercial or financial relationships that could be construed as a potential conflict of interest.

## Publisher’s Note

All claims expressed in this article are solely those of the authors and do not necessarily represent those of their affiliated organizations, or those of the publisher, the editors and the reviewers. Any product that may be evaluated in this article, or claim that may be made by its manufacturer, is not guaranteed or endorsed by the publisher.

## Footnotes

1
www.graphpad.com

## Supplementary Material

Supplementary Figure 1 **(A)** Scatter of microflow cytometry size standards including both fluorescent latex and non-fluorescent silica standards ranging in size from 110 to 1,300 nm. **(B)** Red lines illustrate the division of size ranges of detected EVs described in the text to carry GFP-tagged 72Q Htt*^exon1^* cargo. **(C)** NTA size standards (mean) included 60, 200, and 400 nm polystyrene NIST size standards as well as a 100 nm silica size standard.

Supplementary Figure 2 **(A)** NTA analysis of anti-CD36FITC antibody aggregation. **(B)** Line graph of the time dependent aggregation of anti-CD36-PE antibody **(C)** NTA profiles of anti-CD36-FITC (or PE) antibody aggregation.
