# Supplementary material for: Cysteine String Protein Controls Two Routes of Export for Misfolded Huntingtin
Source: Front Neurosci. 2022 Jan 5;15:762439. doi: 10.3389/fnins.2021.762439 (PMC8766765; doi:10.3389/fnins.2021.762439)
Supplement: Supplementary file 1 [file Data_Sheet_1.PDF]

Supplementary Table 1: MicroFlow Cytometer Settings				
**Platform**	Apogee A50 MP	S/N 0027		
**Parameter**	**Setting**			
Sample Flow Rate	3.01 µL/min			
Pressure	150 units			
Acquisition time	60 sec			
Sample Dilution	25x with PBS			
Volume/well	250 µL			
Sample volume	10 µL			
Diluent volume	250µL			
Event Trigger	LALs unless stated otherwise			
**Channel**	**Laser Power (mW)**	**PMT**	**Gain**	**Threshold**
405nm	75			
488nm	50			
561nm	N/A			
638nm	75			
405-SALS		342	1.0	20
405-LALS		350	1.0	48
405-Red		350	1.0	
405-Green		500	1.0	
488-Green		450	1.0	
488-Orange		550	1.0	
488-Red		580	1.0	
561-Orange		NA	1.0	
561-Red		NA	1.0	
638-Red		550	1.0	
638-Far Red		NA	1.0	
**Beads**	**Product number**	**Lot number**	**Expiration**	
Monitoring	Apogee 1493	CAL0093<br>CAL0095	09/08 2022<br>13/02/2023	

**Supplementary Table 2: LM10 NTA Settings**				
**Platform**	LM10			
**Parameter**	**Setting**			
Software	NTA 3.0 0064			
~Temp (°C)	18-25	**Sample Dilution** 25x		
Camera settings	Type = sCMOS Level =12			
Slider shutter = 600	Slider gain = 350	Shutter/ms = 26.6	Frame rate = 18.8	
Histogram Upper = 8325		Histogram Lower = 0		
Detection Settings	Avg completed tracks samples =3000 <based on sub sample of 40 samples>		Avg completed tracks medias =14 <based on sub sample of 12 samples>	
Threshold = 7	Blur = Auto	Vibration correction applied	Particles per frame = 10-100	
Size distribution - Number weighting - With Percentiles				
Size distribution - Surface area weighting - With Percentiles				
Size distribution - Volume weighting - With Percentiles				
**Beads**	**Product number**	**Lot number**	**Expiration**	
NIST Polystyrene	Thermo Scientific 3000 Series	3060A: Lot40223<br>3200A: Lot 39557<br>3400A: Lot 40720	See lot specific dates;minimum 2 yrs	
Nanosight 100nm Polystyrene size standard	Nanosight stock solutions	N/A	N/A	

Supplementary Table 3: MIFlowCyt / MISEV Compliant Items	
**Requirement**	**Please Include Requested Information**
1.1. Purpose	To assess cellular export capacity of Huntington aggregates using an expressed form of polyglutamine expanded huntingtin (GFP-tagged 72Qhuntingtinexon1) in cells.
1.2. Keywords	microflow cytometry, extracellular vesicle, molecular chaperone, CSPα, DnaJC5, Huntington's disease
1.3. Experiment variables	Conditioned media, expression of polyglutamine expanded huntingtin (GFP-tagged 72Qhuntingtinexon1) and other vectors in CAD neural cells. Specific details in Methods.
1.4. Organization name and address	Hotchkiss Brain Institute<br>Department of Biochemistry and Molecular Biology<br>Cumming School of Medicine, University of Calgary<br>3330 Hospital Dr. N.W.<br>Calgary, Alberta, Canada T2N 4N1
1.5. Primary contact name and email address	Hotchkiss Brain Institute<br>Department of Biochemistry and Molecular Biology<br>Cumming School of Medicine, University of Calgary<br>3330 Hospital Dr. N.W.<br>Calgary, Alberta, Canada T2N 4N1
1.6. Date or time period of experiment	2017 - 2020
1.7. Conclusions	Our data links the molecular chaperone, CSPα, and the packaging of pathogenic misfolded huntingtin into extracellular vesicles for elimination
1.8. Quality control measures	1493 Apogee Bead Mix, NIST 200nm bead standard
2.1.1.1. (2.1.2.1., 2.1.3.1.) Sample description	Conditioned (serum free) DMEM media from CAD neural cells collected 48hrs post transfection.
2.1.1.2. Biological sample source description	Palmitoylated GFP positive PC3 cells (PC3 ATC: CRL-1435) were generated using a palmitoylation signal genetically fused in-frame to the N terminus of EGFP (previously described: Lai et al, 2015).
2.1.1.3. Biological sample source organism description	
2.1.2.2. Environmental sample location	
2.3. Sample treatment description	Conditioned (serum free) DMEM media from CAD neural cells collected 48hrs post transfection.<br>1. Samples were subjected to a single 300xg centrifugation for 5minutes prior freezing (-80°C) and subsequent analysis by micfroflow cytometry or NTA;<br>2. For some samples, prior to microflow cytometry analysis, EVs were treated with Cell Mask Deep Red plasma membrane stain (ThermoFisher C10046) (0.1X final concentration);<br>3. Palmitoylated GFP positive PC3 cells (PC3 ATC: CRL-1435) were generated using a palmitoylation signal genetically fused in-frame

	to the N terminus of EGFP (previously described: Lai et al, 2015 Nature Communications). Cells were permitted to grow to confluency in serum free DMEM (T175flasks, ~20mLs per flask) and EVs collected in the supernatant following a single 300xg centrifugation for 5minutes prior freezing (-80°C).<br>4. Following 300xg centrifugation for 5 minutes, samples from CAD cells were precipitated using ExoQuick (SBI) prior to SDS-PAGE;<br>5. For imaging, EVs were labels with ExoGlow Protein Red (SBI) as per manufacturers instructions and then applied to recipient CAD cells for 24hrs. Cells were washed 3X prior to imaging.
2.4. Fluorescence reagent(s) description	1. Cell Mask Deep Red plasma membrane stain (ThermoFisher C10046), used at a final concentration of 0.1x (optimal concentration defined by titration).
3.1. Instrument manufacturers, model	1. Apogee A50 MicroPlus Microflow cytometer (S/N 0027): Apogee Flow Systems<br>2. LM10 NTA (Malvern Panalytical NanoSight LM10HSBF Nanoparticle Characterization System)
3.3. Instrument configuration and settings	See Tables
4.1. List-mode data files	*Data files will be submitted to http://flowrepository.org
4.2. Compensation description	No compensation
4.3. Data transformation details	
4.4.1. Gate description	Defined by unstained controls
4.4.2. Gate statistics	Data provided as concentration (events/uL)
4.4.3. Gate boundaries	Defined by unstained controls
