# Supplementary figures and images for: Cysteine String Protein Controls Two Routes of Export for Misfolded Huntingtin
Source: Front Neurosci. 2022 Jan 5;15:762439. doi: 10.3389/fnins.2021.762439 (PMC8766765; doi:10.3389/fnins.2021.762439)

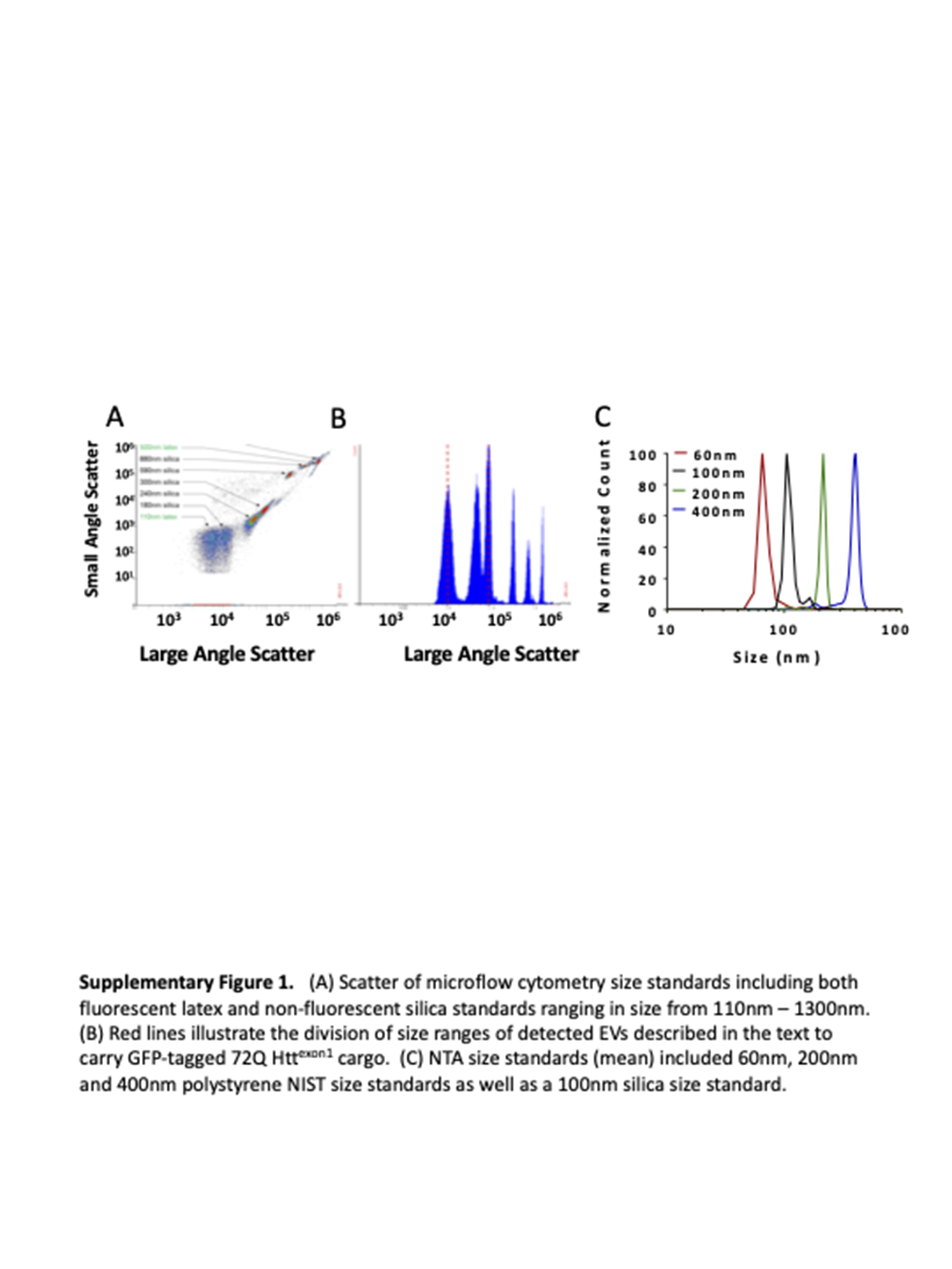

Supplement: Supplementary Figure 1 — (A) Scatter of microflow cytometry size standards including both fluorescent latex and non-fluorescent silica standards ranging in size from 110 to 1,300 nm. (B) Red lines illustrate the division of size ranges of detected EVs described in the text to carry GFP-tagged 72Q Httexon1 cargo. (C) NTA size standards (mean) included 60, 200, and 400 nm polystyrene NIST size standards as well as a 100 nm silica size standard. [file Image_1.tiff]

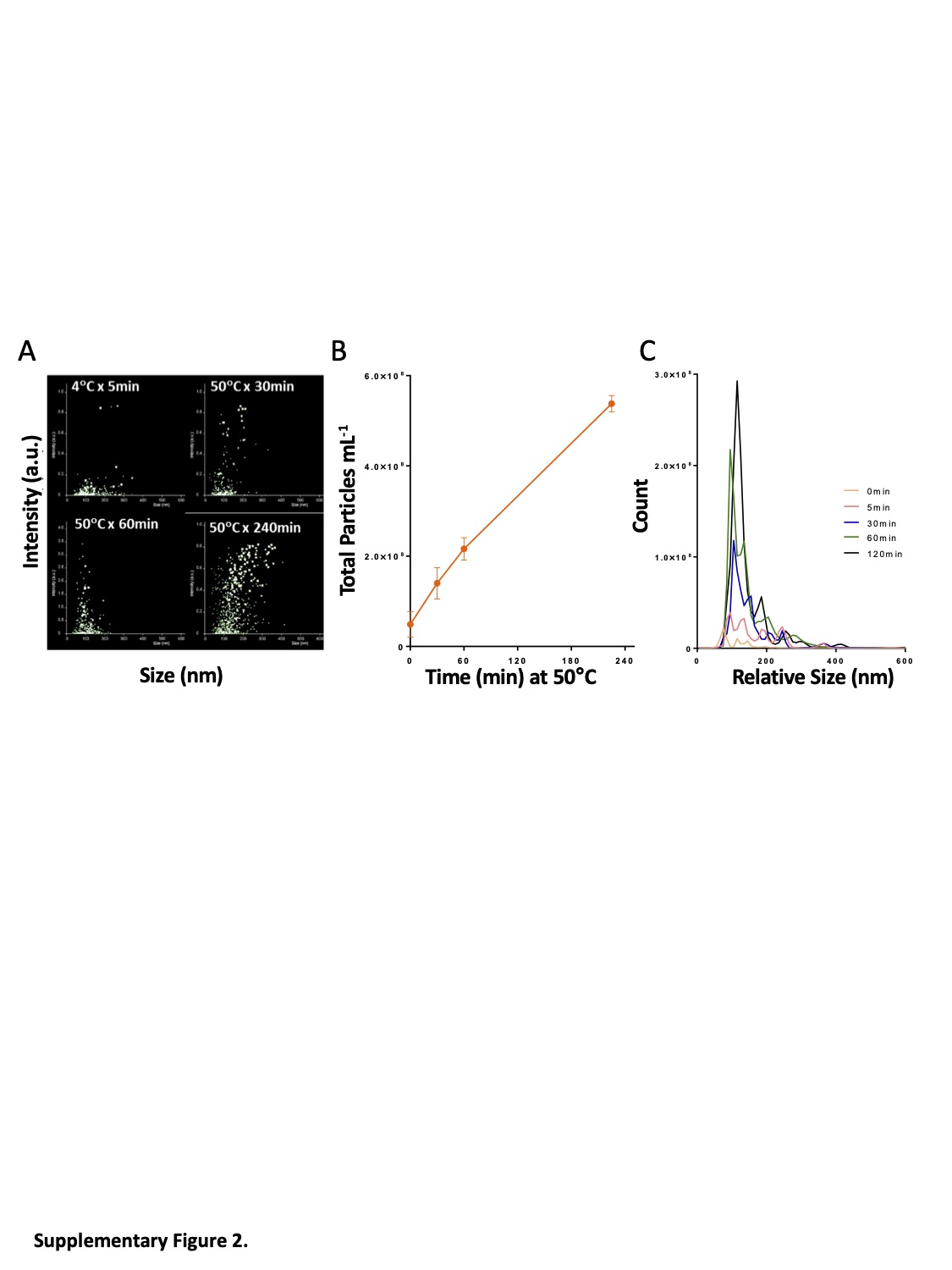

Supplement: Supplementary Figure 2 — (A) NTA analysis of anti-CD36FITC antibody aggregation. (B) Line graph of the time dependent aggregation of anti-CD36-PE antibody (C) NTA profiles of anti-CD36-FITC (or PE) antibody aggregation. [file Image_2.JPEG]
